# Trained immunity in the lung

**DOI:** 10.7554/eLife.104918

**Published:** 2025-08-01

**Authors:** Elina Idiiatullina, Dane Parker

**Affiliations:** 1 Department of Pathology, Immunology and Laboratory Medicine, Center for Immunity and Inflammation, Rutgers New Jersey Medical School Newark United States; Radboud University Nijmegen Medical Centre Netherlands; Indian Institute of Science Education and Research (IISER) India

**Keywords:** trained immunity, innate immune memory, lung, airway, alveolar macrophage

## Abstract

Trained immunity represents a recent concept that elucidates the long-term reprogramming of innate immune cells, enabling them to adapt and respond more effectively to subsequent encounters with diverse pathogens. Initially recognized through the Bacillus Calmette–Guérin vaccine, *Candida albicans* infection, and β-glucan administration, this phenomenon challenges the traditional view that immune memory is exclusive to the adaptive immune system. Trained immunity is characterized by epigenetic and metabolic modifications in innate immune cells that facilitate enhanced responses to infections through mechanisms like chromatin remodeling and altered gene expression. This review focuses on the implications of trained immunity within the lung environment, which is constantly exposed to a plethora of pathogens and environmental irritants. We discuss the roles of various immune cell types, including alveolar macrophages and dendritic cells, in mediating trained immunity and how these adaptations may influence pulmonary insults and disease. Furthermore, we highlight the potential for leveraging trained immunity to enhance vaccine efficacy and develop novel therapeutic strategies for immune-related lung conditions. As research progresses, understanding trained immunity in the lung could pave the way for innovative interventions that improve lung health and resilience against infections.

## Background in trained immunity

**Figure 1.**
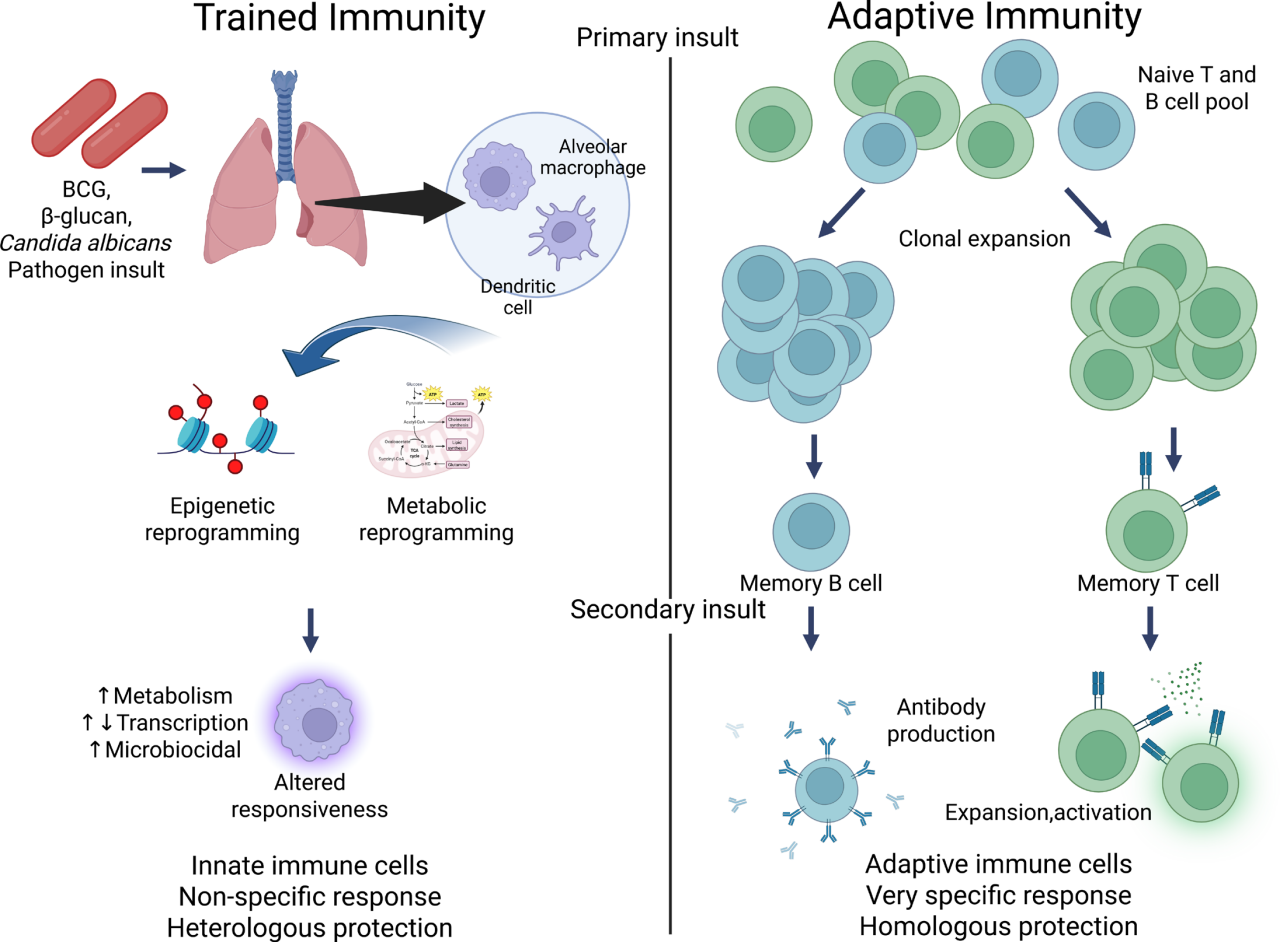
Contrasting trained immunity to adaptive immunity. Figure generated using BioRender.com.

Trained immunity results in the reprogramming of innate immune cells, allowing them to adapt to past encounters with pathogens or inflammatory stimuli. Initially demonstrated in studies on the Bacillus Calmette–Guérin (BCG) vaccine for tuberculosis (Netea et al., 2011; Netea et al., 2020), this phenomenon has been shown to enhance resistance to diverse infections (Garly et al., 2003; Kaufmann et al., 2022a) and even certain cancers (Buffen et al., 2014; van Puffelen et al., 2020). Early evidence of trained immunity was also observed in studies involving *Candida albicans* (Quintin et al., 2012) and its cell wall component β-glucan (Netea et al., 2011; Cheng et al., 2014). The idea of innate immune memory, or ‘trained immunity’, contrasts with the long-standing belief that immunological memory was solely a feature of the adaptive immune system, which includes T and B lymphocytes that produce specific responses to pathogens (Netea et al., 2020; Peignier and Parker, 2020; Hartung and Esser-von Bieren, 2022). Unlike adaptive immunity, which relies on gene recombination for antigen specificity, trained immunity is mediated by epigenetic and metabolic reprogramming of innate immune cells, including monocytes, macrophages, and natural killer (NK) cells (Sun et al., 2009; Foley et al., 2012). These modifications enable innate immune cells to ‘remember’ prior exposures and respond more effectively when confronted with new, unrelated infections. For example, exposure to stimuli such as the BCG vaccine or β-glucan (a component of fungal cell walls) can prime monocytes and macrophages to react more robustly to future infections, whether bacterial, viral, or fungal in origin (Figure 1; Quintin et al., 2012; Cheng et al., 2014; Saeed et al., 2014; Cirovic et al., 2020).

Trained immunity is not exclusive to humans or mammals; it has been observed in a wide variety of species, including plants and invertebrates (Kurtz, 2005; Conrath et al., 2015; Gourbal et al., 2018) that lack adaptive immune systems altogether. In these organisms, trained immunity serves as the primary mechanism for generating immune memory, allowing them to defend against reinfection in the absence of specialized T or B cells. This discovery has challenged the traditional division of the immune system into innate (rapid but nonspecific) and adaptive (slower but highly specific) arms. Instead, it suggests that the innate immune system is capable of a form of memory that allows it to adapt and improve its responses over time, though in a less specific way than the adaptive immune system (Netea et al., 2011).

Mechanistically, trained immunity is driven by epigenetic changes (Cheng et al., 2014; Hole et al., 2019). Epigenetic control of histone modifications facilitates the long-term opening of chromatin (Zhao et al., 2019). After an initial stimulus, specific epigenetic changes and chromatin remodeling allow for altered expression of effector genes upon re-exposure, typically with advantageous outcomes. Trained macrophages exhibit non-permanent histone modifications, such as H3K4 monomethylation (H3K4me), trimethylation (H3K4me3), and H3K27ac (Saeed et al., 2014). These modifications lead to the opening of chromatin at the promoters of pro-inflammatory cytokine genes, including interleukin-6 (IL-6), IL-1β, and tumor necrosis factor (TNF), thereby enhancing protection against re-infection (Quintin et al., 2012). While trained immunity is characterized by heightened inflammatory responses that help eliminate infections more effectively, certain contexts demand a dampened response to prevent excessive inflammation. This phenomenon, known as immune tolerance, represents a distinct regulatory mechanism in which innate immune cells reduce their responsiveness to repeated stimulation, contrasting with the enhanced responsiveness seen in trained immunity (Novakovic et al., 2016). Tolerance can act as a protective mechanism to reduce immunopathology (Medzhitov et al., 2012); however, a drawback of this is the potential for immune paralysis, in which immune cells fail to produce pro-inflammatory mediators, leading to potential susceptibility to secondary infections. These opposing phenomena reflect the immune system’s capacity to adapt dynamically to balance effective defense and tissue homeostasis.

## Induction of trained immunity in lung-resident cells

The induction of innate immune memory in the lung varies significantly depending on systemic versus local exposure. Systemic exposure, such as vaccination or bloodstream infections, influences hematopoietic progenitors in the bone marrow, generating circulating trained immune cells (Netea et al., 2016; Kaufmann et al., 2018). However, the extent to which these cells infiltrate the lung or influence long-lived, embryonically derived alveolar macrophages (AMs) or other resident cells in the airway remains unclear.

Systemic exposures, administered via intravenous or intraperitoneal routes, act over longer intervals—most studies report a delay of 5–7 days for progenitor reprogramming, with secondary challenges occurring anywhere from 2 to 8 weeks later (Kaufmann et al., 2018; Mitroulis et al., 2018). Conversely, local exposure, such as inhaled pathogens or airway-delivered immunostimulants, can directly reprogram lung-resident immune cells, particularly AMs and interstitial macrophages (Yao et al., 2018; Moorlag et al., 2020). These cells play crucial roles in respiratory disease outcomes, but their relative contributions compared to centrally trained innate cells require further investigation (Ginhoux and Guilliams, 2016; Tan and Krasnow, 2016). Local exposures are generally delivered intranasally or via intratracheal instillation, directly targeting lung tissues. In these studies, the time intervals between the primary local insult and the secondary challenge are often shorter—ranging from 3 to 14 days (Yao et al., 2018; Moorlag et al., 2020). This enables the direct assessment of functional reprogramming of lung-resident cells such as AMs.

The interplay between centrally and locally trained innate cells in respiratory pathology is not fully understood. While systemic exposure may enhance circulating immune responses, it is unclear how this translates to protection or pathology within the lung microenvironment (Mulder et al., 2019). Similarly, local exposure may confer protection primarily at mucosal surfaces, but whether this training extends systemically remains an open question (Mantis et al., 2011; Kleinnijenhuis et al., 2012). This distinction between exposure routes and timing is essential as it likely affects the spatial and temporal dynamics of trained immunity within lung tissues. Clearer delineation of these parameters will improve our understanding of how innate immune memory is compartmentalized and its relevance for host defense or immunopathology. Further research is needed to determine how different routes of exposure influence trained immunity in the lung and how these responses shape susceptibility to respiratory infections and inflammatory diseases.

One of the key features of trained immunity is that it not only affects fully differentiated innate immune cells like monocytes and macrophages but also impacts their progenitor cells in the bone marrow. This process, known as ‘central trained immunity’, allows for long-lasting changes in the immune system as the reprogrammed progenitor cells give rise to a new generation of immune cells that inherit these adaptive traits. This central reprogramming ensures that the effects of trained immunity can persist for months to years, extending the period during which the host is better protected against infections or inflammatory conditions (Kaufmann et al., 2018; Mitroulis et al., 2018; Chavakis et al., 2019).

As an extension of central trained immunity, some data suggests that trained immunity may have transgenerational effects (Moore et al., 2019; Berendsen et al., 2020). There is some evidence of this in humans: children of mothers vaccinated with BCG showed reduced mortality (Berendsen et al., 2020). In mouse models, maternal infections or inflammatory conditions during pregnancy have been shown to induce changes in the immune systems of offspring, potentially through epigenetic reprogramming of progenitor cells. Intravenous administration with zymosan or *C. albicans* in parental mice led to progeny with enhanced responsiveness to LPS and protection against infection. This transmission of trained immunity is thought to be due to methylation changes in sperm DNA (Katzmarski et al., 2021). It should be noted in a similar parallel study, no such transmission of training was observed (Kaufmann et al., 2022b). Sperm with altered methylation was also observed after sepsis in mice (Bomans et al., 2018). In the progeny of sepsis mice, only males displayed altered responses, albeit a decrease in cytokine levels, both in plasma and by AM. More recently with SARS CoV-2, children born to mothers with severe COVID-19 were observed to have increased NK cells, γδ T cells, and plasma cytokine levels (Gee et al., 2021). Environmental factors, such as the maternal microbiome, may also play a role in shaping how trained immunity is passed from one generation to the next (Moore et al., 2019; Berendsen et al., 2020; Katzmarski et al., 2021; Katzmarski et al., 2022). This is an area that still warrants further investigation to determine if indeed it occurs and also whether this can impact the pulmonary immune environment.

## Trained immunity and disease

While the mechanisms and effects of trained immunity are well-characterized for bacterial and fungal infections, research is still emerging on its role in other diseases. For instance, there is growing evidence that trained immunity may play a role in chronic inflammatory diseases, cardiovascular disease (Flores-Gomez et al., 2021), and even cancer. In these contexts, the enhanced responsiveness of innate immune cells could influence disease progression, either by promoting inflammation or by boosting the body’s defenses against malignant cells (Bekkering et al., 2014; Buffen et al., 2014; van Puffelen et al., 2020).

This newfound understanding has profound implications for clinical practice. For example, leveraging trained immunity could improve vaccine efficacy, especially in populations where traditional vaccines fail to induce strong adaptive immune responses. Additionally, harnessing the principles of trained immunity could lead to novel treatments for chronic inflammatory diseases, autoimmune disorders, and cancer, by either boosting beneficial immune responses or dampening harmful inflammation (Aaby et al., 1995; Aaby et al., 2006; Aaby et al., 2010; Aaby et al., 2011; Benn et al., 2013; Lund et al., 2015; Biering-Sørensen et al., 2017; Rieckmann et al., 2017; Andersen et al., 2018).

In summary, trained immunity refers to the ability of innate immune cells to undergo long-term reprogramming after encountering pathogens or inflammatory stimuli, resulting in enhanced or altered responses to subsequent challenges. This process, driven by epigenetic and metabolic changes, has been observed across a wide range of species and offers a novel perspective on how the immune system adapts to protect the host. As research into trained immunity progresses, it has the potential to transform our understanding of immune defense and open new avenues for innovative therapeutic strategies. In this review, we specifically focus on the role of trained immunity in the lungs. The lung environment presents unique challenges, being exposed to a constant influx of pathogens and environmental irritants. Understanding how trained immunity operates in the respiratory system could have significant implications for preventing and treating lung infections, chronic inflammatory lung diseases, and even lung cancer. We will explore the latest findings related to trained immunity in the lungs and highlight promising future directions for research in this area, with the ultimate goal of advancing clinical applications in lung health.

## Conceptual framework of trained immunity and tolerance

Trained immunity and innate immune tolerance represent two opposing, adaptive programs of innate immune memory. While trained immunity is defined by hyperresponsiveness to secondary stimuli, tolerance reflects a state of hyporesponsiveness, serving distinct roles in host defense and tissue homeostasis.

Trained immunity leads to an enhanced functional state of innate immune cells following an initial stimulus. This response is non-specific and is driven by epigenetic remodeling (e.g., histone modifications like H3K4me1, H3K4me3, and H3K27ac) and metabolic rewiring, such as increased glycolysis and cholesterol synthesis. These modifications allow trained cells to respond more robustly upon re-exposure to pathogens. For example, in the lung, AMs that have undergone trained immunity exhibit heightened responsiveness to bacterial pathogens, as demonstrated in *Streptococcus pneumoniae* exposure models (Aegerter et al., 2020). Similarly, influenza-induced monocyte-derived AMs (Mo-AMs) can contribute to bacterial defense through IL-6-mediated mechanisms.

In contrast, innate immune tolerance is a suppressive state in which cells reduce their inflammatory output in response to repeated or chronic stimulation. This dampening mechanism is often induced by prolonged exposure to endotoxins, such as LPS. Tolerized macrophages show repressive epigenetic changes at key inflammatory gene loci and diminished production of cytokines like TNF and IL-1β. This LPS-induced tolerance is thought to protect the host by limiting tissue-damaging inflammation (Novakovic et al., 2016). However, it can also result in immunoparalysis, where immune cells become unresponsive to secondary infections, increasing the risk of opportunistic or secondary infections (Winters et al., 2010; Wang et al., 2014; Arens et al., 2016; Shen et al., 2016). These phenomena are not mutually exclusive and may coexist or evolve over time within the same tissue. In the lung, for instance, chronic exposure to inhaled allergens or particulate matter may lead to tolerogenic reprogramming of macrophages, impairing pathogen clearance (Ogger and Byrne, 2021). Meanwhile, acute infections or mucosal adjuvants may induce trained immunity that enhances host defense, though at the potential cost of immunopathology, such as exaggerated inflammation and tissue damage (Coleman et al., 2013; Adami et al., 2018).

Ultimately, the shift toward trained immunity or tolerance depends on multiple contextual factors: nature and dose of the initial stimulus, duration and frequency of exposure, tissue microenvironment and resident cell identity, and host metabolic and immune status. Understanding this dynamic balance is critical in respiratory diseases, where either excessive immune activation or profound immune suppression can worsen outcomes. Therapeutic strategies that selectively harness trained immunity—while avoiding maladaptive hyperinflammation—may offer new approaches for enhancing mucosal protection without promoting chronic inflammatory damage.

## Cell types involved in trained immunity in the airway and the impact on lung infections and immunity

### Macrophages and monocytes

In the airway, several immune cell types, including macrophages and monocytes, play a central role in trained immunity (Table 1; Foster et al., 2007; Quintin et al., 2012; Saeed et al., 2014). This adaptation is crucial for defending the respiratory system against the continuous exposure to bacterial, fungal, and viral pathogens. In response to bacterial infections, particularly in the lung, macrophages and monocytes undergo epigenetic and functional reprogramming. For example, exposure to *S. pneumoniae* alters AMs, leading to long-lasting epigenetic changes that enhance their protective responses during secondary challenges (Aegerter et al., 2020). However, this protection is time-sensitive and can vary depending on the stage of infection (McCullers and Rehg, 2002; Shahangian et al., 2009; Siegel et al., 2014; Sharma-Chawla et al., 2016). This trained immunity has been observed in both mouse and human models of lung infections, underscoring its critical role in maintaining lung health and bolstering immunity against recurrent respiratory threats (Kleinnijenhuis et al., 2012; Quintin et al., 2012; Cheng et al., 2014; Arts et al., 2018).

**Table 1.** Summary of trained immunity studies and lung diseases.

Cell type(s)	Stimulus	Mechanism(s)	Outcomes	Notes	Study
Alveolar macrophages (AMs), monocyte-derived AMs (Mo-AMs)	Sequential infections: influenza virus followed by *Streptococcus pneumoniae*	Influenza leads to depletion of resident AMs through apoptosis, migration, or functional inactivation (‘alveolar macrophage disappearance reaction’). CCR2-dependent monocyte recruitment replenishes the AM niche with Mo-AMs. Mo-AMs undergo IL-6-mediated epigenetic training, enhancing early antibacterial responses.	Improved survival and reduced bacterial burden after secondary pneumococcal infection. Mo-AMs persist but gradually lose protective traits over time.	Trained immunity is transient (~2 months post-influenza).	Aegerter et al., 2020
Resident alveolar macrophages (AMs)	PepO protein from *Streptococcus pneumoniae*	PepO protein stimulates complement C3 secretion and G-CSF production by AMs, without full activation. Establishes a trained phenotype by enhancing innate bactericidal function against unrelated pathogens.	Central-trained immunity established; increased resistance to bacterial pneumonia without inducing systemic inflammation.	Highlights possibility of trained immunity via non-lethal microbial components.	Xu et al., 2024
Tissue-resident alveolar macrophages (TR-AMs)	Intranasal adenoviral vector administration	Local viral infection stimulates CD8+ T cells to produce IFN-γ, which primes TR-AMs to upregulate MIP-2 and KC chemokines. This enhances neutrophil recruitment to the airways during secondary bacterial infections. Training occurs without monocyte input (local imprinting).	Improved early bacterial clearance upon *Streptococcus pneumoniae* challenge.	Provides evidence that viral infections can directly induce trained immunity in TR-AMs.	Yao et al., 2018
Mo-AMs replacing TR-AMs (especially in aged lungs)	Aging process, prior respiratory viral infections (influenza)	Aging leads to impaired TR-AM survival and impaired self-renewal capacity. Viral infections exacerbate depletion. CCR2-mediated recruitment of monocytes leads to replacement by Mo-AMs. Mo-AMs show increased glycolysis and a hyper-inflammatory phenotype compared to TR-AMs, contributing to tissue damage and chronic inflammation.	In aged mice, infections cause more severe lung injury due to predominance of glycolytic, inflammatory Mo-AMs rather than quiescent TR-AMs.	Emphasizes metabolic reprogramming (Warburg effect) and its detrimental effects in elderly lung immunity.	Li et al., 2022
Resident airway macrophages	SARS-CoV-2 infection	Persistent chromatin remodeling around type I interferon (IFN-I) response genes, even after viral clearance. Increased accessibility of IRF and STAT transcription factor motifs. Suggests formation of ‘innate immune memory’ following viral pattern recognition.	Enhanced baseline antiviral state, potential impact on future respiratory infections.	Mechanisms still under investigation; likely involve both direct viral sensing and damage signals (DAMPs).	Lercher et al., 2024; Simonis et al., 2025
Natural killer (NK) cells	Viral infections, BCG vaccination	NK cells acquire memory-like properties after infections. Enhanced IFN-γ, IL-1β, and IL-6 production upon secondary stimulation. Primed for faster and stronger responses.	Improved clearance of respiratory viruses; enhanced responses to secondary challenges.	NK cell-trained immunity impacts airway antiviral defense and broader innate immune memory.	Sun et al., 2009; Romee et al., 2012; Kleinnijenhuis et al., 2014
Dendritic cells (DCs)	Cryptococcus neoformans infection, RSV infection	Exposure leads to epigenetic reprogramming of DCs. Increased IFN-γ and pro-inflammatory cytokine production upon secondary encounters. DC-mediated protection relies on cytokine production like IFN-γ, TNF-α, and IL-17a, as well as STAT1 pathway activation.	DCs play a crucial role in trained immunity and protection against reinfection.	Proper activation of DCs is crucial for protective immunity; impaired DC responses can lose protection.	Hole et al., 2019
Dendritic cells (DCs)	Respiratory syncytial virus (RSV) infection	RSV-triggered TSLP induces epigenetic reprogramming in bone marrow-derived DCs, altering cytokine production and upregulating costimulatory molecules. This leads to an enhanced inflammatory phenotype and exacerbated allergic responses.	RSV-induced trained immunity via TSLP alters immune cell responses and can promote allergic diseases.	Innate immune memory may amplify allergic susceptibility and interfere with appropriate antiviral responses.	Hole et al., 2019; Malinczak et al., 2021
Alveolar macrophages (AMs) and epithelial cells	β-glucan exposure, bleomycin-induced injury	β-glucan primes AMs and epithelial cells via soluble mediators. This leads to enhanced efferocytosis, increased SIRT1 expression, and tissue protection by reducing fibrosis and apoptosis.	β-glucan-induced trained immunity protects against injury and fibrosis, particularly in lung epithelial cells.	Enhanced tissue resilience, reduced apoptosis, and increased resistance to lung fibrosis.	Kang et al., 2024

AMs primarily originate from embryonic precursors and self-renew in the lung under steady-state conditions. However, during infections or inflammatory insults, bone marrow-derived monocytes can be recruited to the lung and differentiate into Mo-AMs, altering the lung’s immune landscape. The functional characteristics of AMs and Mo-AMs differ significantly. Resident AMs maintain immune homeostasis by clearing surfactant and apoptotic cells while exhibiting a tolerogenic phenotype under normal conditions. In contrast, Mo-AMs, which arise during inflammation or after infections like influenza, display distinct transcriptional, epigenetic, and functional profiles (Aegerter et al., 2022; Campo et al., 2024).

Studies show that influenza infection induces long-term changes in lung immunity through the generation of Mo-AMs that can enhance protection against *S. pneumoniae* infection. Following viral clearance and clinical recovery, 1 month post-influenza, mice demonstrate increased protection from *S. pneumoniae* due to Mo-AMs producing elevated levels of IL-6. These Mo-AMs are recruited through a CCR2-dependent pathway and originate from the bone marrow. While these Mo-AMs display surface markers similar to resident AMs, they exhibit a unique functional, transcriptional, and epigenetic profile. In contrast, resident AMs that have experienced influenza remain functionally similar to naive AMs and do not contribute to this protection. However, with time, these Mo-AMs persist in the lung and gradually lose their protective profile (Aegerter et al., 2020). Despite this, their initial presence after influenza infection reshapes the lung’s immune landscape by adding monocyte-derived cells to the AM population, helping to explain how transient viral infections can have lasting impacts on lung immunity. However, the specific effects of such immune training depend on the context. Xu et al., 2024 demonstrated that the *S. pneumoniae* virulence protein PepO acts as a potent inducer of trained immunity in AM. PepO-induced trained macrophages secrete complement C3, which activates peritoneal B lymphocytes, enhancing their bactericidal capacity. Additionally, G-CSF derived from trained macrophages plays a crucial role in shaping central-trained immunity, suggesting PepO’s potential in developing broad-spectrum anti-infection therapies (Xu et al., 2024).

Lung-resident macrophages are a heterogeneous population originating from both embryonic, also known as tissue-resident AM (TR-AM), and bone marrow-derived sources. AMs, the dominant immune cells in the alveolar space, are primarily derived from fetal monocytes during embryogenesis and maintain themselves through local proliferation under steady-state conditions (Ginhoux and Guilliams, 2016; Tan and Krasnow, 2016). In contrast, under inflammatory conditions—such as infection or tissue injury—bone marrow-derived monocytes are recruited to the lung and can differentiate into Mo-AMs, which partially adopt AM-like phenotypes but differ in transcriptional profiles, metabolic programming, and functional responses (Misharin et al., 2017; Scott and Guilliams, 2018; Yao et al., 2018; Woods et al., 2020).

The ‘AM disappearance reaction’ describes the depletion of resident AMs following exposure to inflammatory stimuli, such as infection. This depletion can occur through apoptosis, migration, or functional inactivation, creating a niche that is later repopulated by Mo-AMs. While these recruited cells can transiently enhance protection, their long-term persistence alters immune homeostasis, potentially leading to exaggerated inflammatory responses and impaired tissue repair during subsequent infections (Lobby et al., 2022; Woods and Mutlu, 2025).

Beyond simple replacement, recruited Mo-AMs may adopt alternative activation states that deviate from classical resident AM function. For instance, in models of respiratory viral infection, Mo-AMs exhibit increased glycolytic metabolism and enhanced antigen presentation compared to TR-AMs (Woods et al., 2020). Other phenotypic transitions include pro-fibrotic macrophages contributing to tissue remodeling (Misharin et al., 2017), and inflammatory subsets expressing high levels of TNF and IL-6 (Hou et al., 2021). These phenotypic shifts suggest that Mo-AMs not only differ from embryonically derived AMs in origin but also in their capacity to influence the immunopathological landscape during and after lung inflammation. The plasticity of lung macrophage populations—governed by their origin, microenvironmental cues, and timing of recruitment—is a critical factor in understanding the heterogeneity of trained immunity responses in the lung.

In the context of aging, Mo-AM gradually replace fetal-derived tissue resident AMs (TR-AMs). This replacement is driven by the increased glycolytic and proliferative capacity of Mo-AMs, which outcompete TR-AMs over time. As Mo-AMs accumulate, they adopt a more pro-inflammatory phenotype, which exacerbates the severity of respiratory viral infections, such as influenza. This is particularly evident in elderly individuals, where the increased presence of Mo-AMs contributes to worsened disease outcomes during both primary and secondary viral infections due to heightened inflammatory responses and impaired tissue repair (Li et al., 2022).

There is also evidence that tissue-resident AMs can also play a role in trained immunity. While resident AMs display limited epigenetic changes following influenza A (Aegerter et al., 2020), adenoviral infections can induce memory in AMs independently of monocyte recruitment. These trained resident AMs exhibit an enhanced ability to produce chemokines MIP-2 and KC upon re-challenge with *S. pneumoniae* leading to enhanced neutrophilic responses. This protection is primed by CD8 T cells via IFN-γ signaling (Yao et al., 2018). This contrasts with SARS-CoV-2 that increases chromatin accessibility of type I interferon (IFN-I) transcription factors in airway-resident macrophages. Establishing this innate immune memory required viral pattern recognition and canonical IFN-I signaling, which enhanced subsequent antiviral responses. Additionally, prior SARS-CoV-2 infection reduced disease severity caused by the heterologous influenza A virus. (Lercher et al., 2024). Contrasting with adenovirus infection, a SARS CoV-2 adenovirus vaccine can induce trained immunity in people. Monocytes from vaccinated patients were elevated and had increased activation markers at 3 months and have enhanced proinflammatory cytokine production upon stimulation. It should be noted, however, that these monocytes had higher cytokine levels at rest and epigenetic analyses were not conducted, which might suggest a priming effect over training (Murphy et al., 2023), which might align with a separate study that found no effect (Stevens et al., 2023).

Timing is crucial with secondary bacterial infections following respiratory viruses. Most superinfection studies examine bacterial infections occurring 1–2 weeks after influenza, a period associated with worse outcomes due to immune suppression. However, in the case of influenza-induced Mo-AMs, studies show that at 1 month post-influenza, the lungs demonstrate enhanced protection against *S. pneumoniae* due to the unique functional profile of these Mo-AMs (Aegerter et al., 2020). This suggests that if the host survives the initial high-risk period for bacterial superinfections, influenza can actually confer later-stage protective benefits through trained immunity mechanisms. Understanding how the timing of viral infections influences innate immune memory and protection against secondary infections could inform strategies to reduce post-viral bacterial complications and guide the development of therapeutic approaches.

Environmental microbial triggers, such as LPS, can also induce innate memory in AMs. Intranasal exposure to LPS specifically primes these macrophages for enhanced reactivity during future infections, a process also dependent on IFN-I signaling and metabolic shifts like fatty acid oxidation and glutaminolysis. While this training can improve bacterial clearance, it also comes with risks: trained AMs may increase bacterial loads and contribute to tissue damage during reinfection by amplifying inflammatory responses. For instance, adoptive transfer of trained AMs led to both increased bacterial burden and lung tissue injury, highlighting the complexity of immune responses shaped by microbial exposure (Zahalka et al., 2022). Repeated exposure to harmful stimuli, such as bacterial endotoxin (LPS) or *Pseudomonas aeruginosa*, can train TR-AMs to enhance their defense mechanisms and ability to resolve injury. These trained AMs show increased resistance to cell death and a heightened capacity for efferocytosis, or improved efficiency in clearing cellular debris, promoting tissue healing. A specialized subset of these AMs, characterized by high expression of MerTK, MARCO, and CD163, plays a critical role in reducing lung inflammation and restoring homeostasis, driven by the transcription factor KLF4 (Chakraborty et al., 2023). The adoptive transfer of trained AMs has been shown to reduce inflammation and protect against severe lung injury in models prechallenged with bacterial endotoxin (LPS) or *P. aeruginosa*. This demonstrates that training not only boosts inflammatory responses to pathogens but also enhances tissue repair and resolution of injury. (Chakraborty et al., 2023). This dual role of trained AMs—balancing heightened defense with improved injury resolution—shows the complexity of their reprogramming and its potential therapeutic value in treating inflammatory lung diseases.

IFN-Is have also shown to be important with another bacterial ligand, peptidoglycan. Administration of the murine nasopharynx with either nonviable *Lactobacillus rhamnosus* CRL1505 (NV1505) or its purified peptidoglycan (PG1505) was effective in enhancing resistance to primary respiratory syncytial virus (RSV) infection and secondary pneumococcal pneumonia. Both NV1505 and PG1505 were able to enhance the numbers of AMs producing IFN-β, a key factor in protecting against these infections. Macrophage depletion confirmed that AMs were essential for reducing pathogen loads and preventing lung tissue damage. Notably, while both NV1505 and PG1505 enhanced respiratory protection, their immunomodulatory effects were strain-specific (Clua et al., 2020).

Roles for tissue-resident AMs in trained immunity to primary bacterial challenge have also been identified. AMs trained with *S. pneumoniae* as the primary challenge display distinct metabolic and signaling changes, including increased phosphocreatine and heightened immune gene activity upon re-infection. Specifically, transcriptomic analysis revealed that prior pneumococcal infection resulted in increased expression of immune mediators, such as Cxcl9, while others, including Cxcl14, Ccl22, and Cx3cl1, were reduced. Phagocytosis receptors such as MSR1 (scavenger receptor A) and CLEC12A were upregulated and CD36 and MARCO were downregulated. These changes in immune gene activity differed from those observed after viral infections, where chemokines like CXCL1 and CXCL2 are typically elevated (Guillon et al., 2020). This highlights the context-specific nature of trained immunity in the lung, where different pathogens and stimuli induce varying immune responses. Arafa et al., 2022 expanded on these findings, demonstrating that Mo-AMs recruited during pneumonia recovery underwent profound long-term remodeling, enhancing protection against secondary infections. These modifications persisted for up to 6 months, altering surface markers, transcriptomes, and metabolomes. Mechanistically, IFN-γ and CCR2 were implicated in this process, with IFN-γ produced by CD4^+^ T cells and other cell types playing a pivotal role in AM phenotype changes. CCR2-dependent monocyte recruitment contributed to the early development of these Mo AMs but was not essential for long-term remodeling. This compartmentalized reprogramming was observed primarily in the affected lung lobes, indicating localized immune adaptations (Arafa et al., 2022). A role for AM in *Mycobacterium tuberculosis* infection, particularly regarding vaccine-mediated immunity, has also been detailed. Respiratory mucosal immunization with a viral-vectored TB vaccine alters the airway innate immune landscape, imprinting AMs with enhanced protective functions. This immunization increases interstitial and monocyte-derived macrophages (IM and MdM) in the lungs, enhancing bacterial clearance. AMs in respiratory mucosal-immunized hosts control *M. tuberculosis* more effectively, with lower bacterial counts in bronchoalveolar lavage fluid (BALF) and lung tissues. This innate immune remodeling offers prolonged, T cell-independent protection against TB, emphasizing the potential for targeting airway innate immunity in vaccine strategies (D’Agostino et al., 2020).

### Role for AM in trained immunity towards cancer

Studies suggest that respiratory viral infections, like influenza, can reprogram AMs to develop sustained antitumor immunity in the lungs. Trained AMs infiltrate tumor sites, eliminating tumor cells and resisting immune suppression, a process driven by IFN-γ and NK cells. In human lung cancer, trained AMs are associated with stronger immune responses, suggesting the potential for leveraging trained immunity in lung macrophages for antitumor therapies (Wang et al., 2023b). Trained immunity in myeloid cells using whole beta-glucan particles (WGP) has been demonstrated to inhibit cancer metastasis. WGP-trained macrophages became more responsive to inflammatory and tumor-derived signals, and in mouse models, WGP treatment reduced tumor metastasis and extended survival. The metabolite sphingosine-1-phosphate and mitochondrial fission drove these effects, and blocking these pathways negated the antitumor benefits (Ding et al., 2023).

### Vaccinations that utilize trained immunity and alveolar macrophages

BCG vaccination has been shown to induce trained immunity within AMs. Jeyanathan et al., 2022 discovered that 5 weeks after subcutaneous BCG administration, AMs in vaccinated mice showed increased expression of MHCII and TLR2 compared to controls, indicating a heightened immune readiness. Upon stimulation with *M. tuberculosis* lysate, these AMs produced significantly more IL-6, IL-12p40, MCP-1, and MIP-1α, demonstrating a robust pro-inflammatory response. The metabolic reprogramming of these AMs was notable: BCG-trained AMs exhibited increased glycolysis without major changes in oxidative phosphorylation, a hallmark of trained immunity. Furthermore, BCG-trained AMs proliferated at a higher rate, as indicated by increased BrdU incorporation, but this was independent of their elevated MHC II expression. This glycolytic shift and increased proliferation suggest that BCG vaccination primes AMs for rapid and sustained responses to infection. Interestingly, this memory phenotype developed over time. At 2 weeks’ post-vaccination, AMs did not yet exhibit these trained characteristics, pointing to a time-dependent process likely linked to the dissemination of BCG to lymph nodes. Only viable BCG, not heat-inactivated BCG, induced this trained phenotype, reinforcing the role of mycobacterial viability in shaping immune memory. Beyond the lungs, BCG also induced a trained phenotype in peritoneal macrophages, which similarly exhibited enhanced MHCII expression and increased cytokine production. This study highlights the systemic impact of subcutaneous BCG on macrophage populations, emphasizing its role in promoting trained immunity across different tissue environments, including the lung, by altering both local and systemic macrophage function (Jeyanathan et al., 2022).

Similarly, studies have shown that BCG-induced trained innate immunity (TII) can enhance protection against pulmonary *S. pneumoniae* infection, primarily through increased neutrophil activity in the lungs. This protection is independent of trained circulating monocytes, providing insights for developing vaccines targeting unrelated respiratory bacterial infections (Kang et al., 2023). BCG vaccination at birth has been shown to enhance the effectiveness of formalin-inactivated respiratory syncytial virus (FI-RSV) vaccines in neonatal mice, helping to address the challenge of enhanced respiratory disease (ERD) associated with previous RSV vaccines. ERD, characterized by a Th2-biased response, lung inflammation, and poor protective immune memory, posed a significant barrier to RSV vaccine development. In a study by Wang et al., 2023a, neonatal mice vaccinated with BCG at birth and later immunized with FI-RSV + Al(OH)_3_ showed improved immune responses. Specifically, BCG vaccination induced trained macrophages and lung tissue-resident memory T cells (TRM), as well as specific cytotoxic T lymphocytes (CTLs), while simultaneously suppressing the Th2 and Th17 immune memory responses responsible for ERD. The BCG/FI-RSV + Al(OH)_3_ combination prevented the induction of innate immune tolerance, which was typically observed with FI-RSV + Al(OH)_3_ alone, by promoting trained macrophages instead of tolerant ones. This trained immunity helped regulate lung inflammation and provided enhanced protection against RSV. Notably, the protection against ERD was linked to the activity of lung-trained macrophages and TRM, rather than memory T cells from other organs, like the spleen. This research suggests that leveraging BCG’s ability to promote trained immunity and balanced Th responses could be a promising strategy for developing safer and more effective RSV vaccines for infants (Wang et al., 2023a). Another study showed that in healthy volunteers, BCG vaccination led to a 4–7-fold increase in IFN-γ production and a twofold rise in monocyte-derived cytokines like TNF and IL-1β in response to unrelated pathogens. These enhanced monocytic functions persisted for at least 3 months, with higher expression of activation markers (CD11b, TLR4), driven by the NOD2 receptor and histone 3 lysine 4 trimethylation. BCG also provided T- and B-cell-independent protection in SCID mice against candidiasis, confirming its role in epigenetically reprogramming innate immunity for broad infection protection (Kleinnijenhuis et al., 2012). Furthermore, BCG vaccination was shown to lead to genome-wide epigenetic reprogramming of monocytes and protection against experimental infection with an attenuated yellow fever virus. These changes, indicative of trained immunity, were strongly linked to increased IL-1β production, a key cytokine in trained immunity, rather than specific IFN-γ responses. Genetic, epigenetic, and immunological studies confirmed IL-1β’s central role, showing that BCG induces functional reprogramming and broad protection against unrelated viral infections (Arts et al., 2018).

In the ongoing battle against tuberculosis, MTBVAC, a live attenuated strain of *M. tuberculosis*, has emerged as a promising alternative to BCG. MTBVAC induces trained immunity through significant epigenetic reprogramming in human monocytes, enriching histone methylation marks (H3K4me3) at the promoter regions of pro-inflammatory genes like IL-6 and TNF-α, similar to the effects observed with BCG and β-glucan (Tarancón et al., 2020). MTBVAC not only induces pro-inflammatory cytokines like IL-1β, IL-6, and TNF-α in a dose-dependent manner, but also stimulates IFN-γ, IL-17, and IL-22 in peripheral blood mononuclear cells 7 days after vaccination. This immunomodulatory effect is comparable to that of BCG. Moreover, MTBVAC induces metabolic reprogramming in human monocytes, increasing lactate production and upregulating oxygen consumption rate and extracellular acidification rate, which are critical for the induction of trained immunity. Interestingly, the inhibition of metabolic pathways like glutaminolysis and oxidative phosphorylation significantly impaired the production of IL-6 and TNF-α, underscoring the importance of metabolic rewiring in trained immunity. Additionally, reactive oxygen species (ROS) production, induced by MTBVAC, further contributed to its immune-stimulatory effects.

In vivo studies using MTBVAC-vaccinated mice demonstrated enhanced pro-inflammatory responses upon secondary challenge with LPS, confirming that MTBVAC induces trained immunity similar to BCG. Furthermore, MTBVAC conferred non-specific protection against *S. pneumoniae* in a mouse model of lethal pneumococcal pneumonia. Vaccinated mice showed a 60% survival rate, and the absence of live bacteria in the surviving animals indicated sterilizing immunity. These findings suggest that MTBVAC may offer broad protective benefits beyond tuberculosis, highlighting its potential as a versatile vaccine platform. (Tarancón et al., 2020).

Mucosal vaccination with the recombinant strain rBCGPPE27^PPE27^, derived from *Mycobacterium bovis* BCG, has been shown to boost trained immune responses in mice, offering superior protection against *M. tuberculosis* and unrelated bacterial reinfections. The rBCGPPE27^PPE27^ vaccine promotes the immunometabolic and epigenetic reprogramming of AMs. This vaccine promotes glycolytic metabolism and enhances cytokine production in AMs, key features of trained immunity. Mechanistically, the effectiveness of rBCGPPE27^PPE27^ depends on mTORC2 and hexokinase 1 (HK-1), as the absence of these factors impairs the vaccine’s ability to induce trained immunity in AMs. Notably, mucosal vaccination with rBCGPPE27^PPE27^ was also effective in augmenting responses against unrelated pathogens. When combined with a COVID-19 vaccine, rBCGPPE27^PPE27^ accelerated the development of virus-specific IgG antibodies, increased pseudovirus neutralizing antibodies, and boosted Th1-biased cytokine production by vaccine-specific T cells, outperforming the BCG/CoV-2 vaccine. These findings demonstrate that rBCGPPE27^PPE27^ induces lung-resident memory macrophages and enhances trained immunity through mTORC2- and HK-1-mediated aerobic glycolysis (Peng et al., 2024).

In both mice and humans, BCG and β-glucan stimulation train hematopoietic stem and progenitor cells (HSPCs), increasing myelopoiesis through key cytokines like IFN-γ and IL-1β. BCG vaccination imprints a lasting myeloid bias on HSPCs, regulated by transcription factors HNF1a and HNF1b, leading to the persistent training of CD14^+^ monocytes (Kaufmann et al., 2018; Mitroulis et al., 2018; Cirovic et al., 2020).

Studies on the bacterial extract-based vaccines Broncho-Vaxom (BV) OM-85 and MV130 demonstrate the potential of orally or nasally administered bacterial extracts to induce trained immunity. MV130, a polybacterial mucosal vaccine manufactured by Inmunotek (Spain), is composed of heat-inactivated whole bacteria, with approximately 90% Gram-positive (such as *S. pneumoniae*, *Staphylococcus aureus*, and *Staphylococcus epidermidis*) and 10% Gram-negative species (including *Klebsiella pneumoniae*, *Moraxella catarrhalis*, and *Haemophilus influenzae*). This vaccine provides long-term heterologous protection against viral respiratory infections in mice by modulating the lung immune landscape (Brandi et al., 2022). This vaccine induces a metabolic shift and reprograms both mouse bone marrow progenitors and human monocytes, promoting enhanced cytokine production. MV130’s protective effects are largely innate immune-mediated, as demonstrated by its ability to confer protection against systemic candidiasis in both wild-type and Rag1-deficient mice, which lack functional lymphocytes. Furthermore, the study implicates specific pathways in trained immunity, with metformin used as a pharmacological inhibitor to disrupt this process. Known to inhibit mitochondrial complex I, metformin limits oxidative phosphorylation (OXPHOS) and reduces ATP production, directly impacting glycolytic reprogramming—a key component in the induction of trained immunity in innate immune cells. This study employed metformin to inhibit the metabolic shifts required for trained immunity, ultimately revealing that MV130’s protection against influenza A virus is reliant on these metabolic mechanisms (Brandi et al., 2022). This underscores the essential role of cellular metabolism in sustaining the protective effects of trained immunity and highlights metformin’s use as a tool to probe its metabolic basis.

Similarly, BV OM-85 consists of lyophilized bacterial extracts from pathogens associated with respiratory infections, such as *H. influenzae*, *S. pneumoniae*, *K. pneumoniae* (both pneumoniae and ozaenae subspecies), *S. aureus*, *Streptococcus pyogenes*, *Streptococcus sanguinis*, and *M. catarrhalis* (Salzmann et al., 2022). Administered orally, OM-85 enhances lung macrophage activity (Salzmann et al., 2022), enabling faster IFN responses upon viral infection. In a murine coronavirus (MCoV) model, BV OM-85 treatment led to the accumulation of interstitial macrophages in the lungs, resulting in accelerated IFN signaling and improved lung tissue protection. RNA sequencing showed that BV OM-85-treated lung tissue resembled an intermediate stage between healthy and infected tissue, indicating a faster return to tissue homeostasis. The beneficial effects of BV OM-85 were dependent on early IFN signaling, and adoptive transfer of naive lung macrophages into recipient mice before infection partially mimicked the protective effects of BV OM-85, suggesting the role of macrophages in this innate immune training. Collectively, these vaccines enhance infection clearance and offer heterologous protection by training innate immunity, making them valuable tools for combating recurrent respiratory infections (Brandi et al., 2022; Salzmann et al., 2022).

## Neutrophils: Systemic exposure-induced granulopoiesis and lung immunity

Neutrophils, traditionally regarded as short-lived effector cells, are the most abundant leukocytes in circulation and are the first responders during infection. Emerging evidence suggests that systemic inflammatory stimuli can induce long-term functional changes in neutrophils through a process known as emergency granulopoiesis. BCG vaccination induces both myelopoiesis and granulopoiesis (Kaufmann et al., 2018; Kalafati et al., 2020) as well as functional reprogramming of neutrophils (Moorlag et al., 2020). Trained neutrophils exhibit increased production of ROS, phagocytosis, and sustained inflammatory cytokine production upon secondary stimulation (Moorlag et al., 2020). The enhanced ROS has also been shown to limit the growth of tumors (Kalafati et al., 2020). These functional adaptations are driven by histone modifications, such as H3K4me3 at inflammatory gene loci, and metabolic shifts favoring glycolysis, enabling neutrophils to mount a more robust antimicrobial response (Moorlag et al., 2020). In the context of respiratory infections, trained neutrophils have yet to be shown to improve host resistance to bacterial pathogens. Excessive neutrophil activation may also contribute to tissue damage in inflammatory lung conditions such as acute respiratory distress syndrome (ARDS) and chronic obstructive pulmonary disease (COPD) (Lawrence et al., 2020; Burn et al., 2021). Therefore, while trained neutrophils can enhance pulmonary host defense, their role in immunopathology highlights the need to better understand the balance between protective and detrimental inflammatory responses.

## NK cells

NK cells are traditionally part of the innate immune system but can exhibit memory-like characteristics after initial exposure to certain viral infections. In the context of respiratory infections, NK cells play a critical role in the airway by responding rapidly to reinfection. These cells can release cytokines, such as IFN-γ, which help in the clearance of respiratory viruses and provide enhanced responses upon subsequent exposure (Sun et al., 2009; Romee et al., 2012). In BCG-vaccinated individuals, NK cells have shown enhanced production of pro-inflammatory cytokines, such as IL-1β and IL-6, in response to various pathogens (Kleinnijenhuis et al., 2014). While these responses indicate NK cells may contribute to the heterologous immune effects of BCG, more research is needed to confirm whether similar NK cell-mediated trained immunity responses could enhance protection against respiratory infections. Understanding NK cells’ role in this context could elucidate potential protective mechanisms in the lung, where pro-inflammatory cytokines play a crucial part in early pathogen defense.

## Innate lymphoid cells (ILCs)

ILCs are tissue-resident lymphocytes that orchestrate immune responses by producing cytokines in response to infection and inflammation (Nagasawa et al., 2018; Fol et al., 2024). They are classified into three major subsets—ILC1s, ILC2s, and ILC3s—based on their cytokine profiles and functional roles. Although traditionally considered short-lived effector cells, emerging studies suggest that ILCs can undergo trained immunity-like adaptations, particularly in barrier tissues such as the lung (Ardain et al., 2019; Hoffmann et al., 2021).

ILC2s, which are essential for type 2 immune responses, can be functionally reprogrammed following exposure to inflammatory cytokines such as IL-33 and IL-25 (Zou et al., 2025). After a resting period of 1 month after intranasal administration of IL-33, lungs exhibit more robust responses to repeated allergen challenge. This is also observed with the serine protease allergen from *Aspergillus oryzae* (Martinez-Gonzalez et al., 2016).

Trained ILC2s exhibit enhanced cytokine production upon secondary stimulation, contributing to prolonged immune responses in allergic airway diseases and asthma (Cao et al., 2023). Similarly, ILC1s have been shown to expand and persist following infections, maintaining a heightened capacity to produce IFN-γ, which can influence subsequent immune responses to viral and bacterial pathogens in the lung (Chen et al., 2019; Hsu et al., 2021). While ILC training may enhance protective immunity against respiratory infections, it also poses risks for chronic inflammatory diseases. In asthma, trained ILC2s contribute to sustained eosinophilic inflammation and airway hyperreactivity, highlighting their potential role in driving persistent lung pathology (van Rijt et al., 2016; Kotas et al., 2021). Understanding the mechanisms that regulate ILC training in the lung could provide new therapeutic targets for modulating airway inflammation.

## γδ T cells

γδ T cells represent a unique subset of T lymphocytes that bridge innate and adaptive immunity. These cells recognize conserved microbial antigens and respond rapidly to infection by producing inflammatory cytokines and exerting cytotoxic effects (Hu et al., 2023). Epigenetic changes within the γδ T cell are important for their development (Roels et al., 2020). Both vaccination with MMR and BCG leads to changes indicative of trained immunity in γδ T cells. The MMR vaccine in humans leads to significant transcriptional and functional changes, with increased cytokine production and metabolic pathways, epigenetics were not analyzed (Röring et al., 2024). Three weeks after BCG vaccination in cattle, γδ T cells had enhanced cytokine responses to TLR stimulation that corresponded to increased chromatin accessibility (Röring et al., 2024). Likewise with BCG in humans, γδ T cells showed increased responsiveness to bacterial and fungal stimuli after vaccination (Suen et al., 2024). It would appear that γδ T cells are influenced by prior vaccination in these examples, although their ability to be influenced in the respiratory tract and if this has any functional impact on outcomes is to be determined.

## Implications for respiratory disease and therapeutic targeting

The ability of neutrophils, NK cells, ILCs, and γδ T cells to undergo trained immunity-like adaptations has significant implications for respiratory health. While these responses can enhance host defense against infections, they also contribute to chronic inflammation and tissue damage in diseases such as asthma, COPD, and ARDS. Future research should focus on identifying the regulatory mechanisms that govern trained immunity in these cell types, with the goal of developing targeted interventions that harness protective immunity while minimizing immunopathology.

Understanding the balance between trained immunity and tolerance in lung-resident and recruited immune cells will be essential for designing novel therapies for respiratory diseases. Therapeutic strategies aimed at modulating trained immunity—such as metabolic reprogramming, epigenetic modulation, or targeted cytokine therapies—could offer new avenues for improving immune responses to infections while reducing inflammation-driven lung damage.

## Dendritic cells (DCs)

DCs also play a significant role in trained immunity within the airways (Table 1). Exposure to pathogens like *Cryptococcus neoformans* leads to epigenetic reprogramming of DCs, resulting in increased IFN-γ and pro-inflammatory cytokine production upon subsequent encounters (Hole et al., 2019). While the increased production of IFN-γ suggests an important role for DCs, their significance is underscored by experimental evidence. Specifically, in a study of pulmonary fungal infections, DCs from mice infected with an engineered strain of *C. neoformans* that produces IFN-γ (H99γ) exhibited robust pro-inflammatory responses, characterized by elevated levels of cytokines like NOS2, CXCL9, and IFN-γ, which correlated with controlled fungal burden and reduced disease severity. Conversely, in wild-type (WT) *C. neoformans* infection, where DCs were skewed toward an anti-inflammatory phenotype, disease outcomes were significantly worse, demonstrating that the correct activation of DCs is crucial for protective immunity. Furthermore, when the DC-mediated response was impaired or absent, the trained immunity and protection against reinfection were lost, emphasizing the pivotal role of DCs in maintaining this protective immune memory. The study also highlights the critical role of cytokine production in DC-mediated trained immunity. The elevated levels of pro-inflammatory cytokines (e.g., IFN-γ, TNF-α, and IL-17a) in DCs from H99γ-infected mice, compared to WT-infected mice, were key indicators of the protective immune response. These cytokines drive a Th1-skewed response, with upregulated genes such as NOS2 and CXCL9 supporting an M1-like polarization of the immune system, leading to effective pathogen clearance. Additionally, transcriptomic analysis showed enhanced activation of the STAT1 pathway, further contributing to the pro-inflammatory profile of these DCs, which not only helps clear the current infection but also primes the immune system for faster and more efficient responses upon subsequent infections. Therefore, the enhanced cytokine production and reprogramming of DCs are central to their function in providing long-term immunity, underscoring their pivotal role in trained immunity (Hole et al., 2019).

Early-life RSV infection has been shown to influence systemic immune responses by inducing innate immune memory, specifically through thymic stromal lymphopoietin (TSLP)-mediated changes in DCs (Malinczak et al., 2021). Specifically, RSV-triggered TSLP drives persistent epigenetic reprogramming in bone marrow-derived DCs (BMDCs), altering their cytokine production and upregulating costimulatory molecules, which alters immune cell responses to subsequent exposures. This TSLP-dependent pathway leads to an enhanced inflammatory phenotype in DCs, which, in turn, exacerbates allergic responses, as demonstrated in adoptive transfer experiments. While most outcomes of trained immunity are advantageous, the innate memory in this case shifts the immune system toward a proinflammatory, Th2/Th17-skewed response, which may enhance allergic susceptibility rather than protecting it. Furthermore, TSLP appears to inhibit appropriate antiviral responses, as BMDCs from Tslpr^-/-^ mice mount a more robust IFN-I response to RSV, which is linked to better antiviral activity (Malinczak et al., 2021). The study indicates that innate memory can have detrimental effects, amplifying inflammatory and allergic disease risks rather than conferring antiviral protection (Malinczak et al., 2021).

## Stromal and epithelial cells

Beyond immune cells, structural cells such as epithelial cells and fibroblasts also undergo epigenetic reprogramming in response to infection or inflammation. This reprogramming can perpetuate chronic inflammation, as seen in airway diseases, or alternatively, provide long-term protection against allergic inflammation. For instance, individuals exposed to microbial environments, such as farms, develop anti-inflammatory immune responses (Naik et al., 2017; Ordovas-Montanes et al., 2020; Lechner et al., 2022). However, compared to other cell types in the airway much less has been uncovered as to their role in training immunity. Stimulation of lung epithelial A549 cells with β-glucan or muramyl dipeptide for a day has been shown to enhance IL-6 and IL-8 production upon stimulation with *S. aureus* 5 days later (Chaumond et al., 2023). This response is accompanied by histone modifications, notably the acetylation of histone 3 at lysine 27 (H3K27), indicating a form of epigenetic reprogramming in these cells. Moreover, the application of the ROS scavenger N-acetylcysteine (NAC) prior to β-glucan pretreatment resulted in reduced IL-6 and IL-8 production upon *S. aureus* exposure, highlighting the role of ROS. Additionally, exposure to *Lactococcus lactis* also led to increased IL-6 and IL-8 production in A549 cells upon stimulation with *S. aureus*, correlated with H3K27 acetylation. This work suggests that airway epithelial cells could be reprogrammed but it is still preliminary.

Similarly, bronchial epithelial cells can develop trained immunity upon exposure to microbial components, such as flagellin from *P. aeruginosa* or Pam3CSK4 (Bigot et al., 2020). After the initial 48 h stimulation, the epithelial cell line BEAS-2B produced higher levels of pro-inflammatory cytokines like IL-8 and IL-6 when exposed again 4 days later to fungal pathogens, LPS, or live bacteria, a response mediated by histone acetyltransferase enzymes. The intensity of the secondary cytokine response did not correlate to the level of primary induction. Blocking these enzymes (e.g., with EGCG or BIX01294) reduces the trained immune response, revealing that specific histone modifications are necessary for maintaining memory-like reactivity in airway epithelial cells (Bigot et al., 2020).

Additionally, systemic innate immune training with β-glucan has been found to protect tissues from local injury. In a mouse model, β-glucan pretreatment reduced mortality and lung fibrosis following bleomycin-induced injury (Kang et al., 2024). This protection was associated with increased neutrophil accumulation, enhanced efferocytosis, and histone modifications in AMs and lung epithelial cells. β-glucan also stimulated AMs to produce RvD1, which increased SIRT1 expression in epithelial cells. SIRT1 plays a critical role in cellular resilience, serving as a histone deacetylase that regulates genes involved in anti-oxidative stress, anti-apoptosis, and cellular repair (Kang et al., 2024). In epithelial cells, enhanced SIRT1 expression helps reduce apoptosis by modulating stress response pathways, stabilizing cellular integrity, and limiting excessive inflammation (Kang et al., 2024). SIRT1 accomplishes this by activating key transcription factors, such as FOXO and NRF2, which upregulate antioxidant defenses and repair mechanisms. By deacetylating these transcription factors, SIRT1 decreases ROS levels, thus protecting epithelial cells from oxidative stress-induced damage (Kang et al., 2024). Moreover, SIRT1 is involved in regulating metabolic pathways that support cellular energy balance and survival under injury conditions. Elevated SIRT1 expression in lung epithelial cells of trained mice supports epithelial cell integrity and reduces the likelihood of fibrosis, as the cells can better resist apoptotic and fibrotic signaling cascades. Neutrophil depletion diminished these protective effects, underscoring the critical role of neutrophils in β-glucan’s tissue-protective properties (Kang et al., 2024).

While trained immunity holds promise as a mechanism for enhanced responses to secondary infections and even cancer, conclusive evidence that immune reprogramming directly drives protection is limited by the absence of targeted experiments in many studies. The reliance on correlational data, without specific genetic models, in some cases, that can ablate or modify cell populations, continues to limit our ability to fully establish the causal role of these cells in protection.

This can also be said for roles in stromal cells, with limited data to date. It has been demonstrated that basal cells can retain an inflammatory memory to certain cytokines in the airway, but in the context of trained immunity, this is yet to be delineated (Ordovas-Montanes et al., 2018). Stimulation of lung fibroblast cell lines would indicate prior exposure can increase secondary exposure; however in the one study examining this, cells only received a 24 h rest period that is not sufficient for cells to return to homeostasis (Yap et al., 2022). Endothelial cells have also been shown to have altered cytokine responses after respiratory viral infections as well as epigenetic changes (Lu et al., 2019; Shao et al., 2021), but phenotypic roles in trained immunity have not been shown. Thus, across epithelial and stromal cells there is no in vivo data to suggests role in pulmonary trained immunity.

## Bacterial effects on epigenetics and trained immunity in the lung

Some microorganisms passively induce epigenetic changes in innate immune cells, while others actively alter chromatin modifications to persist in the host. These modifications vary in stability. Histone modifications, like acetylation and phosphorylation, are short-lived and are rapidly induced by signals, influencing chromatin structure and gene transcription. Histone methylation, however, is more stable and can either activate or repress genes (Pereira et al., 2016). DNA methylation is the most durable epigenetic change, leading to long-term gene silencing and immune memory, and can be passed to daughter cells (Pereira et al., 2016). Non-coding RNAs (ncRNAs), such as microRNAs (miRNAs) and long non-coding RNAs (lncRNAs), regulate gene expression by degrading mRNA or interacting with chromatin-modifying enzymes. Together, these epigenetic mechanisms—histone modifications, DNA methylation, and ncRNA regulation—allow microorganisms to reprogram innate immune cells and manipulate host defenses for long-term survival.

For instance, histone modifications, including methylation and acetylation, can alter gene transcription and chromatin accessibility, and pathogens like *P. aeruginosa* and *S. pneumoniae* can induce these changes to evade immune detection and persist in lung tissue (Hamon et al., 2007; Bandyopadhaya et al., 2016). Specifically, *P. aeruginosa* uses quorum sensing molecules, such as 2-aminoacetophenone (2-AA), to regulate host immune responses by suppressing pro-inflammatory cytokines through histone deacetylase 1 (HDAC1)-mediated hypoacetylation of histone H3K18, thereby reducing immune surveillance and enhancing bacterial survival in the host (Bandyopadhaya et al., 2016).

In addition to histone modifications, DNA methylation changes are also implicated in pathogen-driven immune training. For example, *Helicobacter pylori* infection in the gastrointestinal tract has been linked to altered DNA methylation profiles in respiratory-associated immune cells. This pathogen’s epigenetic modifications extend beyond the gut, promoting chronic inflammation and an increased risk of secondary respiratory infections (Maeda et al., 2017). Chronic exposure to *H. pylori* can create an ‘epigenetic field of cancerization’ by inducing aberrant DNA methylation, which not only compromises lung defenses but also primes the respiratory environment for further pathogenic colonization (Maeda et al., 2017). In the context of lung infections, *M. tuberculosis* has evolved to evade immune responses through a histone methyltransferase it secretes, Rv1988, which directly methylates host histone H3, suppressing the transcription of pro-inflammatory genes and facilitating chronic infection (Yaseen et al., 2015).

Collectively, these findings highlight that pathogen-induced epigenetic alterations in lung and airway immune cells, including histone modifications, DNA methylation, and metabolic changes, play a crucial role in shaping respiratory immunity. These adaptations not only enable pathogens to evade initial immune responses but also contribute to a complex landscape of immune memory and tolerance that can either protect against or exacerbate subsequent infections.

## Implications and future directions

Trained immunity in the airway offers a promising therapeutic avenue across a range of lung diseases. By reprogramming innate immune cells—including AMs and monocyte-derived cells—via epigenetic and metabolic rewiring, we can potentially strengthen both antiviral and antibacterial defenses, especially in the context of recurrent respiratory infections. The BCG vaccine, originally developed against TB, has been shown to induce trained immunity beyond its specific target by enhancing pro-inflammatory cytokine responses and providing heterologous protection against unrelated infections (Kleinnijenhuis et al., 2012; Arts et al., 2018). Epidemiological studies support that BCG vaccination correlates with reduced childhood mortality, partially through non-specific effects mediated by trained innate cells (Netea et al., 2020). Vaccine strategies that exploit trained immunity are evolving. Systemic vaccination (e.g., intradermal BCG) induces central training via hematopoietic progenitors, while intranasal vaccination holds unique potential to reprogram lung-resident immune cells directly. Such strategies could reduce dependence on antibiotics, particularly in the face of multidrug-resistant infections. To date, trained immunity has been predominantly studied in myeloid-derived cells. However, epithelial and stromal cells, due to their longevity and direct interface with inhaled stimuli, may also act as non-hematopoietic reservoirs for innate memory.

Despite the promise, key challenges remain. Most evidence for trained immunity relies on correlative data or ex vivo stimulation assays. Robust causal links between trained immunity and disease outcomes in vivo require genetic models. Addressing these gaps is essential for translating basic discoveries into clinically viable strategies.

Nonetheless, the principle of using TII as a bridge to rapid protection remains compelling, particularly in future pandemics where adaptive responses lag behind pathogen spread. In summary, trained immunity offers a flexible and potent mechanism for enhancing host defense, with translational relevance to various lung diseases. Strategic modulation of innate immune memory—via local or systemic vaccination—could transform preventive and therapeutic approaches in pulmonary medicine.

## Conclusion

Trained immunity in the lung enhances infection outcomes and tissue homeostasis by boosting the ability of lung-resident cells, like macrophages and DCs, to respond quickly to pathogens. This leads to faster pathogen clearance, reduced inflammation, and improved tissue repair, maintaining lung health. Trained immunity has been observed with both viral and bacterial pathogens. The potential of trained immunity extends to vaccine development, offering the possibility of long-lasting protection by reprogramming immune cells to react more robustly to future infections. As research progresses, understanding the mechanisms behind trained immunity will be key to developing targeted therapies for infections, chronic lung diseases, and cancer, underscoring its critical role in future healthcare strategies.
